# RP3Net: a deep learning model for predicting recombinant protein production in *Escherichia coli*

**DOI:** 10.1093/bioinformatics/btag003

**Published:** 2026-01-11

**Authors:** Evgeny Tankhilevich, Sergio Martinez Cuesta, Ian Barrett, Carolina Berg, Lovisa Holmberg Schiavone, Andrew R Leach

**Affiliations:** European Bioinformatics Institute (EMBL-EBI), Wellcome Genome Campus, Hinxton, Cambridgeshire, CB10 1SD, United Kingdom; Data Sciences and Quantitative Biology, Discovery Sciences, BioPharmaceuticals R&D, Astra-Zeneca, Cambridge, CB2 0AA, United Kingdom; Data Sciences and Quantitative Biology, Discovery Sciences, BioPharmaceuticals R&D, Astra-Zeneca, Cambridge, CB2 0AA, United Kingdom; Protein Science, Structure and Biophysics, Discovery Sciences, BioPharmaceuticals R&D, Astra-Zeneca, Mölndal, 431 83, Sweden; Protein Science, Structure and Biophysics, Discovery Sciences, BioPharmaceuticals R&D, Astra-Zeneca, Mölndal, 431 83, Sweden; European Bioinformatics Institute (EMBL-EBI), Wellcome Genome Campus, Hinxton, Cambridgeshire, CB10 1SD, United Kingdom

## Abstract

**Motivation:**

Recombinant protein expression can be a limiting step in the production of protein reagents for drug discovery and other biotechnology applications. We introduce RP3Net (Recombinant Protein Production Prediction Network), an AI model of small-scale heterologous soluble protein expression in *Escherichia coli*. RP3Net utilizes the most recent protein and genomic foundational models. A curated dataset of internal experimental results from AstraZeneca and publicly available data from the Structural Genomics Consortium was used for training, validation and testing of RP3Net.

**Results:**

RP3Net achieves an increase in area under the receiver operator curve (AUROC) of 0.15, compared to a baseline model. When experimentally validated on an independent, prospective, manually selected set of 97 constructs, RP3Net outperformed currently available models, with an AUROC of 0.83, delivering accurate predictions in 77% of the cases, and correctly identifying successfully expressing constructs in 92% of cases.

**Availability and implementation:**

The model, along with installation and running instructions, is available under an MIT licence at https://github.com/RP3Net/RP3Net, DOI 10.5281/zenodo.17243498.

## 1 Introduction

### 1.1 Motivation

The production of protein reagents is an essential part of the research and development process in the pharmaceutical and biotechnology industries. In drug discovery it is often a pre-requisite for screening and hit identification (Zanders 2020, Singh *et al.* 2023). In living tissues, the target protein may occur in very small amounts alongside numerous other biomolecules. To be used for high-throughput screening of drug candidates, structural determination and the development of functional assays, the target protein needs to be expressed in a cell culture and purified. The ability to express a protein depends on multiple factors. First and foremost is the protein itself, but other factors include the cloning vector, the species and strain of the host cells, the codon optimization algorithm, the use of tags and fusion proteins and other experimental conditions (Burgess-Brown *et al.* 2008, 2021, Gordon *et al.* 2008, Gräslund *et al.* 2008, Hayashi and Kojima 2008, Structural Genomics Consortium *et al.* 2008; Haacke *et al.* 2009, Raymond *et al.* 2009, Francis and Page 2010, Zhong *et al.* 2015, Cooper and Marsden 2017, Söderberg *et al.* 2019, Chapple and Dyson 2021, Mahajan *et al.* 2021a, b, Strain-Damerell *et al.* 2021, Morão *et al.* 2022, Simm *et al.* 2022, Kurashiki *et al.* 2023, Schütz *et al.* 2023). The choice of these parameters is often influenced by the details of the downstream experiments (Acton *et al.* 2011), making protein production time-consuming and error-prone, and often requiring multiple iterations and much trial and error. The purpose of this work is to develop a deep learning model to predict soluble protein expression in *Escherichia coli* from the construct sequence, thus accelerating the timescales for protein production from months to weeks, cutting costs and reducing environmental impact.

**Figure 1:**
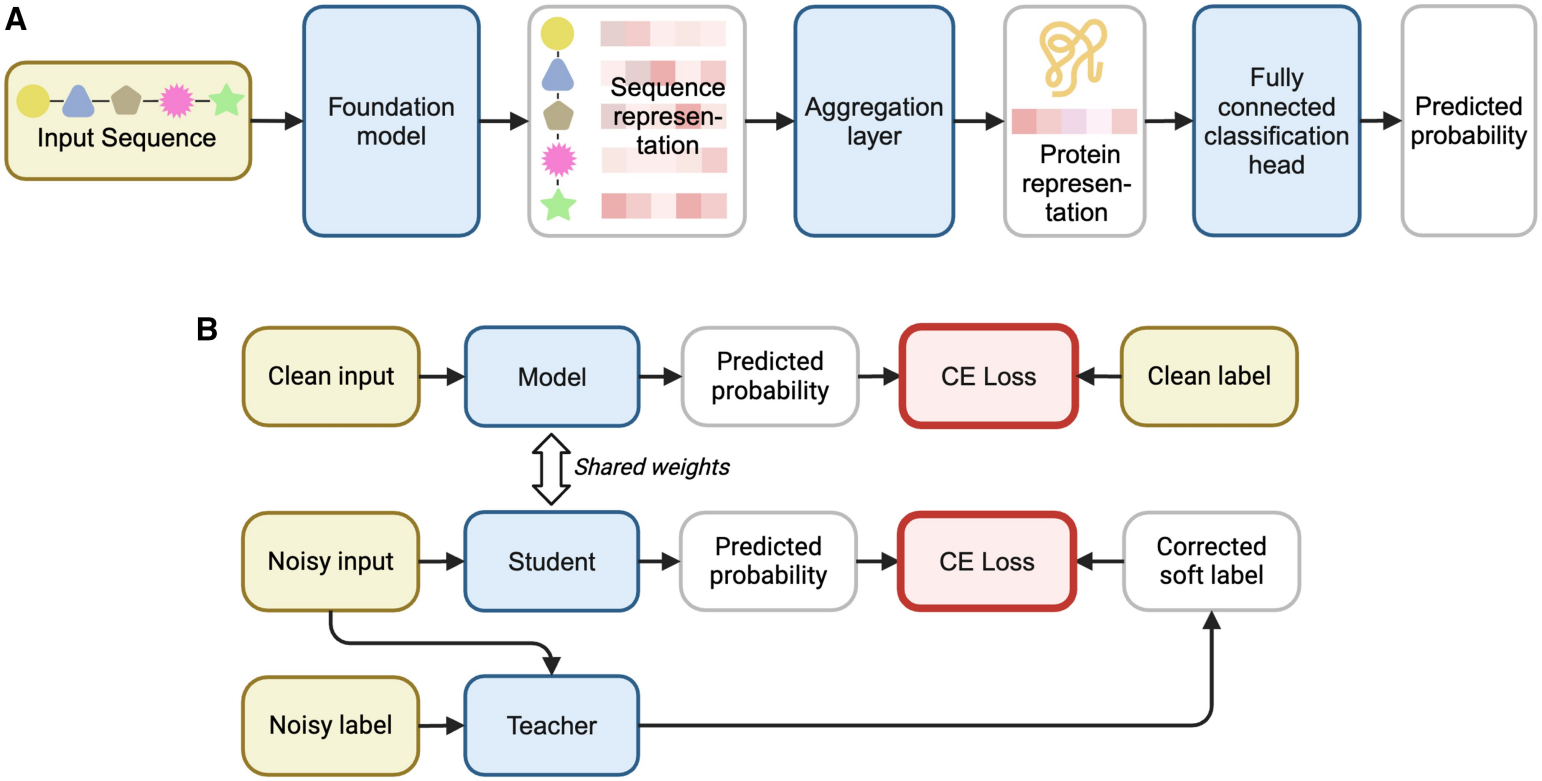
(A) Architecture diagram of RP3Net. The input biological sequence is encoded by the foundation model to obtain a sequence representation, where each residue/codon/nucleotide is represented by a vector. The aggregation layer builds a global protein representation vector from the sequence representation. The predicted probability of successful recombinant expression of the protein in *E. coli* is computed by the fully connected classification head from the protein representation. (B) Training with meta label correction on a mixture of clean and noisy data. The standard training set-up, where the model loss on clean inputs and labels is minimized with gradient descent, is shown in the top row. A special ‘teacher’ model is trained to predict the corrected labels from the noisy input and labels. These corrected labels, along with noisy inputs, serve as inputs for training the ‘student’ model. The latter model has the same architecture and weights as the ‘clean’ model. The bi-level optimization algorithm that makes sure that the corrected labels do not deviate from the (unknown) clean labels relies on using the cross-entropy (CE) loss. Model components with trainable weights are shown as blue boxes. Training data are shown as yellow boxes. Images generated with BioRender.com.

A recombinant protein production experimental pipeline involves several steps, including construct design, cloning, small-scale expression screening, progression of expressing constructs to large-scale purification and quality control (Edfeldt *et al.* 2024). Small-scale soluble expression screening, shown in Fig. 1, available as supplementary data at *Bioinformatics* online, is crucial for assessing whether to progress the construct to large-scale production. First, cells are transfected with vectors (e.g. plasmids) carrying the cloned DNA of the protein of interest (Step 1). Recombinant protein production is performed in deep well format (Step 2). Cells are spun down and lysed (Step 3). The lysate contains the total amount of protein produced. After an additional centrifugation step the soluble protein is found in the supernatant and the insoluble material is discarded in the pellet (Step 3). Soluble protein is captured in a one-step purification using the histidine tag and immobilized metal affinity chromatography (IMAC, Step 4) (Sulkowski 1985). The soluble protein yield and correct size are typically assessed by performing denaturing gel electrophoresis (sodium dodecyl sulphate–polyacrylamide gel, SDS–PAGE) where yield and size are compared to a protein standard (Step 5). The yield can be estimated by quantifying the amount of the target protein compared to the protein standard in the stained gel, using densitometric analysis. At this stage, it is important to record both positive (produced) and negative (failed to produce) experimental outcomes (Step 6). Throughout the rest of this publication, unless stated explicitly, terms ‘protein production’ and ‘protein expression’ refer to this step of the experimental pipeline. Constructs that pass this small-scale screening are then typically progressed to large-scale purification and further downstream applications.

Protein and DNA foundation models (FMs) have become ubiquitous tools for predicting structural and functional protein properties from amino acid and/or nucleic acid sequences (Ji *et al.* 2021, Rives *et al.* 2021, Brandes *et al.* 2022, Elnaggar *et al.* 2022, Lin *et al.* 2023, Nguyen *et al.* 2023, Zhou *et al.* 2023, Outeiral and Deane 2024). These FMs are typically trained on large corpora of sequences, such as UniRef (Suzek *et al.* 2015, Bateman *et al.* 2023), GenBank (Benson *et al.* 2013, Sayers *et al.* 2024), MGnify (Richardson *et al.* 2023) or BFD (Jumper *et al.* 2021) (Table 1, available as supplementary data at *Bioinformatics* online). During training a portion of the sequence is masked out, i.e. each residue is replaced with a special ‘blank’ character. The training objective then becomes to reconstruct the masked portion of the input or ‘fill in the blanks’. This technique, referred to as language modelling task, originates from natural language processing (Devlin *et al.* 2018).

**Table 1:** RP3Net training and architecture configurations.

RP3Net model	Aggregation	FM weights	Meta label correction
A	Mean	Frozen	No
B	STP	Frozen	No
C	STP	Fine-tuned, LoRA	No
D	STP	Fine-tuned, LoRA	Yes

ESM (Rives *et al.* 2021, Lin *et al.* 2023, Hayes *et al.* 2025), ProtBert (Elnaggar *et al.* 2022) and ProteinBert (Brandes *et al.* 2022) are examples of protein FMs; DNABert (Ji *et al.* 2021, Zhou *et al.* 2023) is a popular DNA FM. These models, except for ProteinBert, are based on transformer deep learning architecture (Vaswani *et al.* 2017) with different number of layers, feature dimensions and other details. ProteinBert uses convolutional layers for sequence embedding and global attention for global protein properties. HyenaDna (Nguyen *et al.* 2023) is another DNA FM that uses a different architecture, whereas CaLM (Outeiral and Deane 2024) is a transformer-based model that uses codons as inputs.

The intermediate layers of foundational models yield a residue-level sequence representation that can be used to predict the protein property of interest, such as secondary or tertiary structure, binding affinity, fluorescence, thermodynamic stability, solubility, etc. (Rao *et al.* 2019, Dallago *et al.* 2021, Jumper *et al.* 2021, Rives *et al.* 2021, Thumuluri *et al.* 2022, Wang and Zhao 2022, Lin *et al.* 2023, Li *et al.* 2024, Xiong *et al.* 2024, Hayes *et al.* 2025). The experimental datasets that describe these properties typically contain orders of magnitude fewer entries when compared to the sequence corpora. This scarcity of experimental datasets often makes it unfeasible to train large foundational models from scratch for predicting protein properties. Such models are usually pre-trained with the language modelling objective on the large corpora first, and then further trained to predict the property of interest using the smaller dataset (Rao *et al.* 2019, Dallago *et al.* 2021, Li *et al.* 2024). This final training step is referred to as fine-tuning if the FM weights are updated, or downstream training if these weights are frozen and extra layers are trained to predict the property of interest. Recombinant Protein Production Prediction Network (RP3Net) follows the latter architectural blueprint by encoding the biological sequence with a foundational model, feeding this encoding through an aggregation layer to obtain a global representation for the entire construct, and then applying a fully connected classification head to compute the predicted probability of recombinant expression in *E. coli* as a binary outcome (Fig. 1A).

Although in theory, a soluble protein production fine-tuning dataset could be designed and experimentally generated from scratch, in practice it would be too time-consuming and expensive. Moreover, there already exist publicly available datasets of protein expression that contain the results of experiments worth millions of dollars and representing years of lab work (Gabanyi *et al.* 2011, Berman et al. 2017, Protein production data from the SGC 2022). For training and evaluating RP3Net, internal AstraZeneca (AZ) small-scale expression screen data are combined with datasets from the Structural Genomics Consortium (SGC), specifically their sites in Stockholm (Gräslund *et al.* 2008, Savitsky *et al.* 2010) and Toronto (Protein production data from the SGC 2022, Edfeldt *et al.* 2024). The experimental pipeline for generating data from AZ and SGC Stockholm has been already discussed above, see Fig. 1, available as supplementary data at *Bioinformatics* online, Step 6. SGC Toronto captures the results of large-scale protein purification.

### 1.2 Existing work

A number of models that predict soluble expression from construct sequence have been published in recent years. Most of these systems use datasets derived from the Protein Structure Initiative (PSI) compendium, sometimes referred to as TargetTrack (Gabanyi *et al.* 2011, Berman et al. 2017). PSI was an experimental research effort run across multiple laboratories in 2000–17, with the objective of determining protein structures and depositing them in the Protein Data Bank (PDB) (Berman *et al.* 2000, Burley *et al.* 2023). This dataset records the pipeline position, i.e. the experimental stage where the work was terminated, for each target and construct. For example, if a construct was selected and cloned, but could not be expressed, its pipeline position would be recorded as ‘cloned’. For another construct that has been selected, cloned, expressed and purified, but could not be crystallized, the pipeline position would be ‘purified’, etc. One limitation of using TargetTrack data in this work is that in this dataset a genuine inability to express the construct under given experimental conditions can be confused with stopping to pursue the construct for other reasons (for example there being another well-behaving construct for the same target). Different labs that have provided data for TargetTack were using different experimental pipelines: sometimes small-scale expression screening as shown in Fig. 1, available as supplementary data at *Bioinformatics* online, but also large-scale purification results as SGC Toronto, or the results of running SDS-PAGE on unpurified cell lysate.

NetSolP (Thumuluri *et al.* 2022) uses PSI data to evaluate multiple transformer-based models available at the time for predicting soluble expression. NetSolP outputs two scores: solubility and ‘usability’, the latter being a combined predictor of solubility and the ability of a protein to be expressed.

It is important to make a distinction between solubility as a general physical property of the protein, which can be measured, for example, as peak concentration in the solution, and the ability to achieve soluble expression of the protein under given experimental conditions, which is a binary outcome that is modelled in this work. A protein that is generally soluble could still fail to express, for example because it is toxic for the host cells, or because the chaperones that are required for forming the correct structure are missing, or due to other reasons. Solubility is thus a necessary but insufficient condition for soluble recombinant protein production.

PLMC (Xiong *et al.* 2024) and SADeepCry (Wang and Zhao 2022) also use data derived from TargetTrack and a Transformer architecture but output the pipeline position given the construct sequence. PPCPred (Mizianty and Kurgan 2011), PredPPCrys (Wang *et al.* 2014), Crysalis (Wang *et al.* 2016), and DCFCrystal (Zhu *et al.* 2021) are examples of older, simpler models that predict pipeline position, trained on various subsets of TargetTrack. SoluProt (Hon *et al.* 2021) uses a different PSI-based dataset with a Gradient Boosted Machine model (Friedman 2001) and global features based on relative amino acid frequencies, predicted physicochemical properties, similarity to *E. coli* proteome and output of various other bioinformatics tools to predict soluble expression.

CamSol (Sormanni *et al.* 2015, Sormanni *et al.* 2017) is a well-established relative solubility prediction tool for libraries of similar protein sequences. There are many other solubility predictions that use deep neural networks, such as GPSFun (Yuan *et al.* 2024) and PLM_Sol (Zhang *et al.* 2024). A few methods exist for modelling expression and solubility of human antibodies, but their experimental protocols differ substantially from *E. coli*-based expression analysed in this work (Zhang *et al.* 2022, Basafa *et al.* 2024).

## 2 Materials and methods

### 2.1 The dataset

Protein production results from AZ, SGC Stockholm and SGC Toronto were used for training and evaluation of the models. AZ and SGC Stockholm report the results of small-scale protein expression testing, after one purification step. In the AZ dataset, the outcome is reported as an expression yield category, manually estimated by the scientist who has expressed the protein. In addition, for a subset of constructs, an estimate of the absolute concentration value in milligrams per litre is provided. The estimate is obtained by comparing the size and intensity of the band on the SDS-PAGE gel for the protein of interest with the band for the reference protein of known concentration. The amino acid sequence of the construct includes affinity and solubility tags; DNA sequences are available for a subset of constructs.

The dataset from SGC Stockholm contains genetic sequences, with tags, annotated with categorical outcomes.

For the bulk of the SGC Toronto data, the outcome is reported as a pipeline position, similarly to PSI/TargetTrack. Importantly, there is no dedicated stage for expression screening: ‘cloned’ is immediately followed by ‘purified’. Although it can generally be assumed that a protein must be expressed before it can be purified, sometimes producing at larger scale (expression volume) can rescue a construct that failed to yield soluble protein at small scale, and vice versa. For a small subset of SGC Toronto data, small-scale expression screening outcome is also provided as a categorical variable, similarly to SGC Stockholm. Genetic sequences are available for a subset of observations, and tags are included in the constructs.

**Figure 2:**
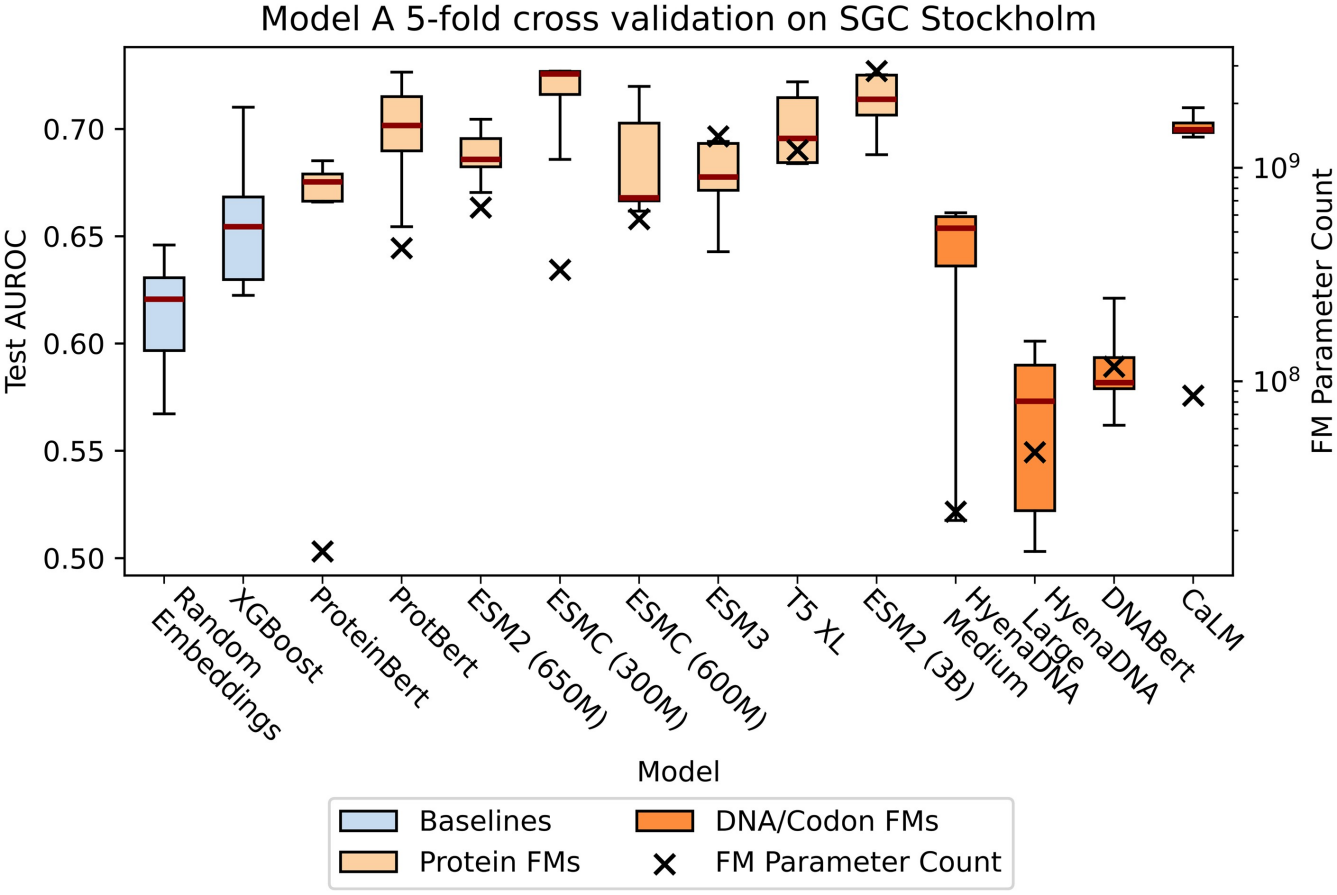
Performance of RP3Net with fixed foundation model (FM) weights and mean pooling (Model A) on SGC Stockholm, along with FM parameter count. On the left *y*-axis, each boxplot shows the area under AUROC of Model A with a particular FM, evaluated on SGC Stockholm test data, with five-fold cross-validation. On the right *y*-axis, black crosses show the number of trainable parameters of the FM, in log scale. ESM2 (650M) was selected for further analysis based on performance, consistency, parameter count and licencing restrictions. ‘Random embeddings’ means using random residue embeddings instead of an FM.

Graphical overview of the datasets is shown in Fig. 2, available as supplementary data at *Bioinformatics* online. There are a total of 67 055 unique sequences, covering 5077 target proteins. Publicly available datasets are significantly larger than the internal AZ dataset, SGC Toronto being the largest. Datasets vary in terms of number of constructs per target, availability of genetic sequences versus protein sequences and imbalance between positive and negative outcomes.

To normalize the data across multiple sources and to compute the outcome imbalance, the labels were converted to binary form, with ‘True’ indicating successful production and ‘False’ failed production. For AZ, this binary outcome was computed based on the existing category annotation, estimate of the absolute concentration and manual re-annotation. For SGC Stockholm, the binary outcome was derived directly from the existing category annotation. For SGC Toronto, it was derived from the pipeline position. The details of this procedure are given in the Supplementary methods, available as supplementary data at *Bioinformatics* online.

### 2.2 The model

RP3Net encodes biological sequences using a foundational model, applies an aggregation layer to generate a global protein representation, and then passes this representation through a classification head to produce the final score (Fig. 1A). Two types of aggregation layer were tested in this work: mean pooling and set transformer pooling (STP). For notation, assume that for a sequence of length *N*, the output of the foundational model for each residue *i* is represented with a column vector $x_{i}^{F}$ from a *d*-dimensional space, $x_{i}^{F}\in R^{d\times 1}$. The matrix representation for the entire protein, $X^{F}$, is obtained by stacking these residue representations along the sequence dimension: $X^{F}\in R^{d\times N}$. In this notation, mean pooling, which is just averaging all these residue representations, can be written as


(1) XMG=1N∑i=1NxiF. #

The advantage of mean pooling is that it is simple to interpret and fast to compute. The disadvantage is that it does not have any trainable parameters, or weights, so all training must happen upstream, in the foundational model, or downstream, in the classification head. STP is an example of an aggregation layer with trainable weights. Here, the global representation is the result of performing multiheaded attention (MHA) (Vaswani *et al.* 2017, see Supplementary text, available as supplementary data at *Bioinformatics* online), with a seed vector, $w^{S}\in R^{d}$, as a query, and residue representations $X^{F}$ as keys and values. Thus,


(2) XSTPG=MHA(wS,XF,XF).#

The meta label correction (MLC) (Zheng *et al.* 2021, Taraday and Baskin 2023) framework is used for training the model on predicted small-scale expression labels, derived from large-scale expression labels when the former are not available in the dataset. This framework utilizes a larger, noisy, poor-quality dataset to augment the training process of the model that would normally use only a smaller, clean, high-quality dataset. A separate, ‘teacher’ model is trained to predict the corrected soft label from the noisy data and labels. These corrected labels, along with the clean inputs and labels, are used to train the original model, which in this set-up is referred to as the ‘student’ model (Fig. 1B). Comprehensive mathematical details of the MLC algorithm are given in the Supplementary text, available as supplementary data at *Bioinformatics* online.

### 2.3 Experimental validation

The target set for experimental validation of the model was curated to include viable human drug targets and to exclude proteins that are well known from literature to be successfully expressed. We made sure that neither the protein itself, nor its close homologs, have been deposited in the PDB (Berman 2000, Burley *et al.* 2023). We have also excluded the target from the validation set if it was referenced from ChEMBL (Zdrazil *et al.* 2024). OpenTargets (Ochoa *et al.* 2023) was used to check for viability of a drug target. Twenty thousand human proteins from UniProt (Bateman *et al.* 2023) were narrowed down to 454 viable targets. These targets were further curated manually, to have distribution across different target classes and avoid too many DNA-binding proteins. In the end, 46 targets were selected for experimental validation.

Two full length constructs were created per target: one with a TEV-cleavable 6His tag and a GS linker at the N-terminal (MHHHHHHENLYFQGS…) and another one with a GS linker and a 6His tag at the C-terminal (…GSHHHHHH). Soluble production of the full-length constructs was predicted with RP3Net. For the targets where both full-length constructs were predicted to fail to be produced, trimmed constructs were generated by iteratively removing residues from N- and C-termini, with a minimum construct length of 50. Trimmed constructs that were predicted to express successfully were included in the experimental validation set. Seventy per cent of the set comprised constructs that were predicted to be produced, with the remaining 30% as negative controls. A total of 97 constructs were available for expression testing after taking into account cost constraints and cloning errors. The details of the experimental procedures are given in the Supplementary text, available as supplementary data at *Bioinformatics* online.

## 3 Results and discussion

### 3.1 RP3Net with fixed FM weights outperforms decision trees with global protein features

The RP3Net architecture with fixed FM weights and mean pooling (Fig. 1A) was used for selecting the best performing FM. This architecture is denoted as Model A (Table 1). The models were trained and evaluated on the SGC Stockholm dataset, with five-fold cross-validation. SGC Stockholm was used because this dataset is of medium size, compared to AZ, which is much smaller, and SGC Toronto, which is much larger. This dataset is also the only one of the three that provides DNA sequences for all the constructs.

A gradient boosted decision tree, XGBoost (Friedman 2001), with global protein features as inputs was used as a baseline model. As shown in Fig. 2, Model A with any protein FM outperforms the baseline model. Out of all the tested DNA and codon FMs, only CaLM (Outeiral and Deane 2024) shows better results than the baseline. A plausible explanation is due to the datasets used for pre-training the FMs: CaLM was pre-trained on coding sequences from European Nucleotide Archive, whereas other DNA FMs were pre-trained on a mixture of coding and noncoding sequences.

RP3Net performance also varies depending on the training data subset that was used, sometimes dramatically. For example, for the more consistent FMs, such as ESM2 (650M) and CaLM, the difference between the best and the worst runs is 0.03 and 0.01, respectively, whereas for HyenaDNA Medium the difference is 0.14.

The number of trainable FM parameters is used to indicate the resource requirements for fine-tuning the FM (compute time, memory). The FM for subsequent evaluation was chosen based on the pragmatic trade-off between performance, data availability, training complexity and licencing constraints (Table 1, available as supplementary data at *Bioinformatics* online). We selected ESM2 with 650 million parameters. The simple Model A training protocol, applied to this FM, achieves an average increase in area under the receiver operator curve (AUROC) of 0.03, compared to the baseline model.

### 3.2 Performance on different data sources reveals dependency on dataset size

Model A performance on SGC Stockholm dataset can be improved by replacing the mean pooling aggregation layer with a more sophisticated STP (Lee *et al.* 2019, Buterez *et al.* 2022). This configuration is denoted as Model B. The main difference between mean pooling and STP is that, whereas the former just takes an average across the sequence, giving each residue the same weight, STP uses context-dependent weights for residue representations, by computing multiheaded attention (MHA) (Vaswani *et al.* 2017), see Supplementary text, available as supplementary data at *Bioinformatics* online) between a special parameter, called the seed vector, and the output of the FM. The seed vector is updated during training with gradient descent, along with the rest of the model parameters.

Model B gives an AUROC improvement of 0.02 over Model A when trained and evaluated on SGC Stockholm (Table 2). The performance of Model B on other data sources varies with an AUROC of 0.59 on the AZ dataset and of 0.84 on SGC Toronto. A plausible reason for this variation is dataset size. Training Model B on the combined AZ and SGC Stockholm data improves evaluation of AZ to 0.73, which is almost the same as evaluating the same model on SGC Stockholm. Adding SGC Toronto to the training data does not improve the evaluation results significantly for any data source.

**Table 2:** Results of evaluating different RP3Net models trained on different data sources versus the baseline model and third-party predictors.

Model	Trained on	Tested on	AUROC	Accuracy	Recall	Precision
NetSolP solubility		AZ	0.64	0.61	0.44	0.64
NetSolP useability		AZ	0.64	0.57	0.13	**0.87**
B	AZ	AZ	0.59	0.52	**0.85**	0.50
B	SGC Stockholm	AZ	0.73	0.65	0.63	0.65
B	SGC Stockholm, AZ	AZ	0.73	0.66	0.72	0.63
B	SGC Stockholm, SGC Toronto, AZ	AZ	0.71	0.66	0.50	0.72
C	SGC Stockholm, SGC Toronto, AZ	AZ	0.72	0.66	0.52	0.71
D	SGC Stockholm, AZ, SGC Toronto	AZ	**0.74**	**0.70**	0.71	0.69
D	SGC Stockholm, SGC Toronto	AZ	0.69	0.67	0.49	0.75
NetSolP solubility		SGC Stockholm	0.48	0.66	0.14	0.42
NetSolP useability		SGC Stockholm	0.39	0.68	0.00	0.00
Baseline	SGC Stockholm	SGC Stockholm	0.62	0.62	0.39	0.40
A	SGC Stockholm	SGC Stockholm	0.70	0.63	0.74	0.45
B	SGC Stockholm	SGC Stockholm	0.72	0.63	0.74	0.45
B	SGC Stockholm, AZ	SGC Stockholm	0.73	0.62	**0.78**	0.45
B	SGC Stockholm, SGC Toronto, AZ	SGC Stockholm	0.70	0.60	0.64	0.42
C	SGC Stockholm, SGC Toronto, AZ	SGC Stockholm	0.68	0.62	0.56	0.43
D	SGC Stockholm, SGC Toronto, AZ,	SGC Stockholm	**0.77**	0.73	0.54	0.59
D	SGC Stockholm, SGC Toronto	SGC Stockholm	0.72	**0.75**	0.51	**0.66**
B	SGC Stockholm, SGC Toronto, AZ	SGC Toronto	0.75	0.87	**0.12**	0.27
C	SGC Stockholm, SGC Toronto, AZ	SGC Toronto	**0.76**	**0.88**	0.08	**0.33**

Numbers highlighted in bold indicate the highest value of the metric achieved when testing on a given dataset.

### 3.3 Meta label correction with purification data yields a 0.04 increase in AUROC on SGC Stockholm

Both Model A and Model B are fine-tuned on soluble protein expression data with frozen parameters of the FM. Unfreezing these parameters (Model C) and training on the full dataset leads to overfitting: perfect performance is quickly achieved on the training dataset (AUROC≈1.0), but on the validation and test sets the AUROC remains below 0.75. Training Model C on individual data sources also leads to overfitting, as expected.

This could be explained by the fact that the datasets contain the results of slightly different experiments. The SGC Toronto dataset reports results of large-scale purification, whereas both AZ and SGC Stockholm report small-scale expression testing captured with one-step purification. Although the exact experimental conditions, materials and methods used for purifications were not available during model development, it is safe to assume that the SGC Toronto conditions are quite different from the small-scale expression testing. A natural question arises: given the construct sequence from SGC Toronto, and its binary purification result, what would be the result of small-scale expression testing this construct under the conditions of SGC Stockholm or AZ?

We address this within the MLC framework (Zheng *et al.* 2021, Taraday and Baskin 2023), where a large, noisy dataset is used to aid training the model on a small, clean set. Rather than adding the noisy data directly to the training set, a special model is trained to predict the corrected label from the noisy input and noisy label. This is referred to as the ‘teacher model’. The corrected labels are used to train the ‘student model’, along with clean inputs and clean labels (Fig. 1B). In our set-up, SGC Toronto large-scale purification dataset is used to train the teacher model, and a union of SGC Stockholm and AZ small-scale expression data are used to train the student model.

Using MLC with large-scale purification data (Model D) achieves in AUROC of 0.74 on AZ dataset. This is an improvement of 0.01 compared to the second-best result of Model B on AZ data. When evaluating Model D on SGC Stockholm, AUROC reaches 0.77, which is an improvement of 0.04 over the next-best result. We have also observed that the MLC model is more robust across different sequence clusters—training, validation and testing—than other models, which tend to over-fit the training data. The MLC framework thus allows utilizing large-scale purification data to improve modelling of small-scale expression testing, whereas simple transfer learning (Model B or Model C trained on all sources) fails to achieve that outcome.

Adding a relatively small number of constructs from AZ to the training set has a larger impact on model performance than using MLC, when evaluated on SGC Stockholm data. This points to a potential further improvement of the model in the future, by better accounting for the difference in experimental conditions between the labs that have provided the data. Using MLC still results in the highest AUROC when evaluating on all data sources, which justifies using this approach.

### 3.4 Prospective experimental validation of the model shows AUROC of 0.83

To establish the utility of RP3Net for drug discovery projects, in addition to the normal train–validate–test model development loop, we have conducted prospective model evaluation in a real-life scenario. A set of 46 proteins was curated from the human proteome to include viable drug targets, whilst avoiding proteins with prior published evidence of successful expression. We started by generating two full length constructs per target (with a 6-His affinity tag placed at the N- or C-termini) and running RP3Net on them. If both constructs were predicted not to express, we generated trimmed constructs, ran these through the model, and, if they were predicted to express, included them in the dataset. This resulted in a total of 97 constructs for the experimental validation dataset, eight of which were generated by the trimming process. The constructs were cloned and expressed in *E. coli* at the AZ protein production facility. Forty-nine per cent of the constructs passed small-scale expression screening, including one-step purification by affinity chromatography (Fig. 3 and Table 2, available as supplementary data at *Bioinformatics* online).

The performance of RP3Net Models B and D was compared with the baseline model, and two third-party predictors: SoluProt (Hon *et al.* 2021) and NetSolP (Table 3; Table 2, available as supplementary data at *Bioinformatics* online). The highest AUROC of 0.83 is achieved by RP3Net Model D. This is 0.08 better than the best third-party predictor (NetSolP useability). RP3Net Model D showed an accuracy of 0.77 when the score cut-off of 0.5 was used, and accuracy of 0.81 with the cut-off set to 0.79.

**Table 3.** Results of experimental validation of RP3Net, the baseline model and third-party predictors.

Model	Trained on	AUROC	Accuracy	Recall	Precision
Soluprot		0.64	0.57	0.83	0.57
NetSolp solubility		0.66	0.60	0.79	0.59
NetSolp useability		0.75	0.57	0.23	**0.86**
Baseline	SGC Stockholm	0.67	0.65	0.58	0.71
RP3Net B	AZ	0.69	0.65	0.88	0.62
RP3Net B	SGC Stockholm	0.81	0.72	**0.96**	0.65
RP3Net B	AZ, SGC Stockholm,	0.77	0.71	**0.94**	0.66
RP3Net B	AZ, SGC Stockholm, SGC Toronto	0.76	0.69	0.65	0.74
RP3Net D	AZ, SGC Stockholm, SGC Toronto MLC	**0.83**	**0.77**	0.92	0.73
RP3Net D	SGC Stockholm, SGC Toronto MLC	**0.83**	**0.77**	0.87	0.75

Numbers highlighted in bold indicate the highest value of the metric achieved when testing on a given dataset.

To assess the impact of having AZ data in the training set, the performance of Model D trained on the amended set that excludes AZ data was compared with the model that was trained on the full set. The small AZ dataset has very little impact on the model performance during experimental validation; both AUROC and accuracy figures are identical, with small differences in precision and recall with score cut-off of 0.5.

For the subset of eight trimmed constructs, RP3Net D shows an accuracy of 0.5 with score cut-off of 0.5, and accuracy of 0.62 with score cut-off of 0.79. This could be an artefact of the small evaluation set, or that RP3Net does not consider if sequences will fold into stable protein domains. Curiously, the trimmed constructs that did result in soluble protein also contained degradation products (Fig. 3, available as supplementary data at *Bioinformatics* online). Using the score cut-off of 0.5, the model predicts all trimmed constructs to express, whereas in fact only four out of eight were expressed successfully.

Performance on trimmed constructs could thus be considered an area for improvement. However, considering the small number of trimmed constructs, and the model accuracy (0.77) and precision (0.73) on the larger experimental validation set, it could be argued that an experimental scientist would still find the modelling results helpful. An ‘overconfident’, high recall, model that predicts too many positives, which are then partly confirmed in the laboratory, is preferrable to a model that misses out constructs that would have expressed in the lab. Model precision could be increased, at the expense of recall, by increasing the score cut-off threshold.

## 4 Conclusion

The recombinant production of proteins can require multiple experimental rounds of trial and error. To improve the efficiency of such experiments, we have developed RP3Net, an AI model of heterologous protein expression in *E. coli*. RP3Net predicts the results of protein expression as a binary outcome. It was built using the latest foundational models and was trained using a combination of internal experimental results from small-scale AZ expression screens, and publicly available data from the SGC. Using an STP aggregation layer and MLC with large-scale purification data enables RP3Net to achieve state-of-the-art performance both on the take-out data from SGC Stockholm and AZ. RP3Net has been experimentally validated on a manually selected set of constructs for viable human drug targets and outperformed third-party predictors on that set as well. Ablation studies show that there is no single method that achieves a large performance increase, but rather many small incremental improvements.

This work also underscores the need for large and well-curated datasets of soluble protein expression and for the scientific community to agree on how the data should be captured following the FAIR principles, and to establish a protein production ontology (Wilkinson *et al.* 2016, Edfeldt *et al.* 2024). Unfortunately, in the field of protein production, there is not yet an equivalent of the PDB for structural biology. Significant time in this project was spent on data curation.

The modelling results may also be further improved by making the model more aware of the experimental conditions, such as *E. coli* host strain, induction methods, time and temperature at which various experimental stages were performed, buffer formulations, etc, This information is largely missing from the available datasets.

RP3Net is already deployed and used by the protein scientists at AZ. This publication and the accompanying code repository at GitHub make the model available to the wider research community, both in industry and in academia (Edwards *et al.* 2025).

## Supplementary Material

btag003_Supplementary_Data

## Data Availability

The model code is available under an MIT licence at GitHub, https://github.com/RP3Net/RP3Net, DOI 10.5281/zenodo.17243498. The dataset used for training and evaluating the model, except for AZ data, is available at https://ftp.ebi.ac.uk/pub/software/RP3Net. The AZ data are subject to AZ confidentiality policies.
