# Supplementary material for: RP3Net: a deep learning model for predicting recombinant protein production in Escherichia coli
Source: Bioinformatics. 2026 Jan 11;42(1):btag003. doi: 10.1093/bioinformatics/btag003 (PMC12857573; doi:10.1093/bioinformatics/btag003)
Supplement: btag003_Supplementary_Data [file btag003_supplementary_data.zip › supplements/rp3net_bioinformatics_supp_text.docx]

RP3Net: a deep learning model for predicting recombinant protein production in *Escherichia coli*

# Supplementary information

Evgeny Tankhilevich, Sergio Martinez Cuesta, Ian Barrett, Carolina Berg, Lovisa Holmberg Schiavone and Andrew R Leach

Table of Contents

[Supplementary text 1](#_Toc214634989)

[Binarization of experiment outcomes in the dataset 1](#_Toc214634990)

[Cross validation 2](#_Toc214634991)

[The baseline model 2](#_Toc214634992)

[Multi-headed attention 2](#_Toc214634993)

[Meta Label Correction Algorithm (MLC) 2](#_Toc214634994)

[RP3Net implementation and training 4](#_Toc214634995)

[Experimental procedures for construct expression. 4](#_Toc214634996)

[Supplementary figures 5](#_Toc214634997)

[Supplementary tables 9](#_Toc214634998)

[References 10](#_Toc214634999)

# Supplementary text

## Binarization of experiment outcomes in the dataset

Historical AZ small-scale expression screening results were reported as concentration range estimates: "0 to 1", "1 to 10", "10 to 20", "20 to 50", and "above 50" mg/L. The data were converted to binary outcomes as follows. Results in "0 to 1 mg/L" concentration range were annotated as False (not produced). Results within "10 to 20", "20 to 50", and "above 50" mg/L were annotated as True (produced). Results in the "1 to 10" range were handled in a special manner. The experiments that had an estimate of absolute concentration were annotated as either True or False by comparing this value with the threshold of 3.5 mg/L. The experiments where the absolute value had not been estimated were re-annotated manually, by re-examining the captured image of the SDS-PAGE gel.

AZ data that were collected after April 2023 do not contain manual estimates of the concentration range. Instead, each experiment outcome is manually classified by the scientist into three categories, based on the SDS-PAGE gel: "Passed", "Not passed" and "Ambiguous". Outcomes belonging to the "Passed" category were annotated as True, and those belonging to "Not passed" and "Ambiguous" categories as False.

SGC Stockholm reports small-scale soluble expression screening outcomes with manual numeric qualitative annotations by the lab scientist: 0 – no soluble expression, 1 – low soluble expression, 2 – medium, 3 – high and 4 – very high soluble expression. Outcomes from categories 0 and 1 were annotated as False, and with categories 2 and above – as True. Small-scale soluble expression results for SGC Toronto were annotated in the identical manner.

For the SGC Toronto data with pipeline position outcome, results marked with "cloned" were annotated as False, and those with "purified" and beyond - as True.

## Cross validation

To avoid bias towards any particular protein sequence motifs, five-fold cross validation was performed. All constructs were clustered using MMseqs2, commit 25688290 (Steinegger and Söding 2017). The affinity and solubility tags were removed from the constructs, and the remaining "target" sequences were clustered. The following command line was used:

mmseqs easy-cluster -c 0.6 --min-seq-id 0.4 --threads 16 -s 7.5 rp3net_no_tags.fasta rp3net_clustered

Sequence clusters that contain the AZ results recorded after 1st of September 2023, as well as the constructs used for the experimental validation, were grouped together to form the test set. The remaining constructs were divided into five cross validation subsets, such that each cluster is entirely contained within a single subset. The cross validation was performed on Model A with SGC Stockholm data, and the worst performing data split was chosen for subsequent model development and reporting.

Although the clustering procedure above follows multiple peer-reviewed papers (Jumper *et al.* 2021; Zhu *et al.* 2021), in theory it can still lead to data leakage between training and validation set. We believe data leakage not to be a significant issue for RP3Net training, because without MLC (models A, B and C) it overfits, achieving significantly higher performance on training set than on validation set, reaching AUROC of 1.0 on training set for model C (***Figure S4***). If there was significant leakage between training and validation sets, model performance on these data sets would have been expected to be similar.

## The baseline model

A gradient-boosted decision tree (XGBoost v2.1.3) (Friedman 2001) was used as a baseline model. The input features for the tree were generated by analysing the sequences with ProtParam, as well as predicting global protein properties with Schrodinger API v2021-2 (Sankar *et al.* 2022), DisEMBL v2.0 (Linding *et al.* 2003) and RaptorX (Wang *et al.* 2016). The full list of features and methods used to compute them is given in ***Table S3***. For tools that take protein sequence alignments as input, those were built against the Uniprot database downloaded in February 2016, clustered at 20% cutoff (uniprot20_2016_02) (Mirdita *et al.* 2016).

## Multi-headed attention

Computing the MHA involves weight matrices $W_{h}^{Q},W_{h}^{K},W_{h}^{V}$ and $W^{O}$ for queries, keys, values and outputs, respectively, where e $h=1..H$, and $H$ is the number of heads:

$$\begin{aligned} \begin{matrix} \text{MHA}\left( w^{S},X^{F},X^{F} \right) & =\left[ \text{concat}\left( A_{1},A_{2},\ldots,A_{H} \right)W^{O} \right]^{\top}, \\ A_{h} & =\text{softmax}\left( \frac{Q_{h}K_{h}^{\top}}{\sqrt{d_{a}}} \right)V_{h}, \\ Q_{h} & =w^{S\top}W_{h}^{Q}, \\ K_{h} & =X^{F\top}W_{h}^{K}, \\ V_{h} & =X^{F\top}W_{h}^{V}. \\ & \end{matrix}\#\left( 3 \right) \end{aligned}$$

Here, the matrices $W_{h}^{Q}\in\mathbb{R}^{d\times d_{a}}$are used to project $w^{S}$ into $d_{a}$-dimensional space, $W_{h}^{K}\in\mathbb{R}^{d\times d_{a}}$ and $W_{h}^{V}\in\mathbb{R}^{d\times d_{a}}$ – to project $X^{F}$into $d_{a}$-dimensional space, and the matrix $W^{O}\in\mathbb{R}^{hd_{a}\times d}$ – to project the concatenated single head attention outputs back to the $d$-dimensional space. These matrices, as well as the seed vector $w^{S}$, are updated during training. The number of heads, *H*, as well as the inputs and outputs dimension, *d*, and the attention dimension, $d_{a}$, are hyperparameters, that are chosen to maximise model performance on the validation dataset. Matrix transposition, denoted by ⊤, is required to keep the inputs and outputs in column form.

Multi-headed attention is used for the STP aggregation layer in this work, as outlined above. It is also an important part of the transformer architecture, that underpins most of the foundation models.

## Meta Label Correction Algorithm (MLC)

The Meta Label Correction (MLC) (Zheng, Awadallah and Dumais 2021; Taraday and Baskin 2023) framework utilises a larger, noisy, poor-quality dataset to augment the training process of the model that would normally use only a smaller, clean, high-quality dataset. A separate, “teacher” model is trained to predict the corrected soft label from the noisy data and labels. These corrected labels, along with the clean inputs and labels, are used to train the original model, which in this setup is referred to as the “student” model (Fig. 2B in the main text).

Formally, we can denote the clean dataset as $D\equiv\{X,y\}$, where *X* are the inputs and *y* are the labels. The noisy dataset can be denoted as $\tilde{D}\equiv\{\tilde{X},\tilde{y}\}$, and the corrected labels – as $y^{\text{c}}$. The student model that predicts the probability of the clean label *y*, based on the clean input *X*, is denoted $p_{w}\left( X \right)$: $P\left( y|X \right)\sim p_{w}\left( X \right)$, where *w* are the trainable parameters. In the normal deep learning framework this model is trained by minimising the loss function $\mathcal{L}\left( w \right)$between the true labels and the predicted labels over the clean dataset:

$$\begin{aligned} w^{*}={arg min}_{w}\mathcal{L}\left( w \right),\#\left( 4 \right) \end{aligned}$$

For binary labels that take values of 0 and 1, and cross-entropy (CE) loss, we have

$$\begin{aligned} \begin{matrix} \mathcal{L}\left( w \right) & \mathcal{\equiv L}\left( p_{w}\left( X \right),y \right)\equiv CE\left( y,p_{w}\left( X \right) \right) \\ & =y\times log\left( p_{w}\left( X \right) \right)+\left( 1-y \right)\times log\left( 1-p_{w}\left( X \right) \right) \end{matrix}\#\left( 5 \right) \end{aligned}$$

Simple transfer learning would work by $\tilde{X}$ and $\tilde{y}$ and for $X$ and $y$, respectively, in equations (4) and (5). Instead, in the MLC framework, the noisy labels $\tilde{y}$ are replaced by the corrected labels $y^{\text{c}}$, modelled by the teacher model, based on the noisy sequences and the noisy labels: $P\left( y^{\text{c}}|\tilde{X},\tilde{y} \right)\sim q_{\alpha}\left( \tilde{X},\tilde{y} \right)$, with parameters $\alpha$. The loss function $\tilde{\mathcal{L}}$ between the corrected labels and the noisy input is obtained by substituting the teacher model in place of $y$ in the equation (5):

$$\begin{aligned} \tilde{\mathcal{L}}\left( w,\alpha\right)\equiv CE\left( q_{\alpha}\left( \tilde{X},\tilde{y} \right),p_{w}\left( \tilde{X} \right) \right).\#\left( 6 \right) \end{aligned}$$

The optimal parameters of the student model w* now depend on the parameters of the teacher model α:

$$\begin{aligned} w^{*}\left( \alpha\right)={arg min}_{w}\tilde{\mathcal{L}}\left( w,\alpha\right).\#\left( 7 \right) \end{aligned}$$

The optimal value $\alpha^{*}$ needs to be determined, such that the corrected labels $y^{c}$ are indeed meaningful in the context of the student model, or, in other words, that the student model trained on the noisy data with corrected labels performs well on the clean data. This can be done by substituting the optimal value of $w$ defined by equation (7) in the equation (4):

$$\begin{aligned} \begin{matrix} \alpha^{*} & ={arg min}_{\alpha}\mathcal{L}(w^{*}\left( \alpha\right)). \end{matrix}\#\left( 8 \right) \end{aligned}$$

Equations (7) and (8) form the bi-level optimisation problem, that jointly determines the parameters of the teacher and the student models.

In the context of this work, the clean data is a union of the AZ and SGC Stockholm data sets, and the noisy data is SGC Toronto with pipeline position labels. The noisy dataset is thus several times larger than the clean one. On each step of the algorithm several gradient steps through the noisy data (Eqn. 7) are followed by a single step through the clean data (Eqn. 8). The number of noisy steps per single clean step is a hyperparameter, denoted as *k*. Putting it all together, we get **Algorithm 1** for computing $w^{*}$and $\alpha^{*}$.

The teacher parameters $\alpha$ at step $t$ are updated by computing the gradient $g_{\alpha}^{(t)}$ of the clean loss $\mathcal{L}$ with respect to (w.r.t) $\alpha$. This gradient can be approximated by a formula involving the gradient of the clean loss w.r.t student parameters $w$ at step $t+1$, $g_{w}^{\left( t+1 \right)}$, and the matrices of second derivatives (Hessian matrices) of the noisy loss w.r.t $w$ and $\alpha$ at previous steps, $H_{w\alpha}^{\left( \tau\right)}=\frac{\partial^{2}}{\partial w\partial\alpha}\tilde{\mathcal{L}}\left( w^{\left( \tau\right)},\alpha^{\left( \tau\right)} \right)$:

$$\begin{aligned} g_{\alpha}^{\left( t \right)\top}\approx-\eta_{w}g_{w}^{\left( t+1 \right)\top}\sum_{\tau=t-k+1}^{t} \left( 1-\eta_{w} \right)^{t-\tau}H_{w\alpha}^{\tau}.\#\left( 9 \right) \end{aligned}$$

This assumes that gradients are represented as column vectors. For the special case of k=1, the sum in equation (9) is reduced to just $H_{w\alpha}^{\left( t \right)}$; $g_{\alpha}^{\left( t \right)\top}=-\eta_{w}g_{w}^{\left( t+1 \right)\top}H_{w\alpha}^{t}$.

| **Algorithm 1** Bi-level optimisation of teacher and student model parameters via stochastic gradient descent | | | |
| --- | --- | --- | --- |
| **Input:** Clean and noisy datasets $D$ and $\tilde{D}$; number of training steps *T*; initial parameters $w^{(0)}$, $\alpha^{(0)}$ ; learning rates $\eta_{w}$ , $\eta_{\alpha}$, number of noisy steps per clean step k. | | | |
| **Output:** Optimised parameters $w^{(T)}$, $\alpha^{(T)}$. | | | |
| 1 | **for** *t = 0, ..., T – 1* **do** | | |
| 2 |  | $\left\{ X,y \right\}\leftarrow Sample\left( \mathcal{D} \right); \{\tilde{X},\tilde{y}\}\leftarrow Sample\left( \tilde{\mathcal{D}} \right)$ // sample the minibatches of clean and noisy data | |
| 3 |  | $w^{\left( t+1 \right)}\leftarrow w^{\left( t \right)}-\eta_{w}\nabla_{w}\tilde{\mathcal{L}}\left( w^{\left( t \right)},\alpha^{\left( t \right)} \right)$ // update *w* by descending the noisy loss w.r.t. *w* | |
| 4 |  | **if** t mod k = k − 1 **then** | |
| 5 |  |  | $g_{\alpha}=\nabla_{\alpha}\mathcal{L}\left( w^{\left( t+1 \right)}\left( \alpha\right) \right)$ // unroll $w^{t+1}$and approximate the gradient of the clean loss w.r.t. $\alpha$ |
| 6 |  |  | $\alpha^{\left( t+1 \right)}\leftarrow\alpha^{\left( t \right)}-\eta_{\alpha}g_{\alpha}$ // update $\alpha$ by descending the clean loss w.r.t. $\alpha$ |
| 7 |  | **else** | |
| 8 |  |  | $\alpha^{\left( t+1 \right)}\leftarrow\alpha^{\left( t \right)}$ |
| 9 |  | **end if** | |
| 10 | **end for** | | |
|  |  | | |

The CE loss allows for efficient computation of the Hessian $H_{w\alpha}$, by expressing it point-wise as a product of Jacobians, and averaging over the minibatch:

$$\begin{aligned} H_{w\alpha}=\frac{1}{N}\sum_{i=1}^{N} \left[ J_{w}\left( i \right) \right]^{\top}\left[ J_{\alpha}\left( i \right) \right], \#\left( 10 \right) \end{aligned}$$

Here, $J_{w}\left( i \right)$ is the Jacobian (matrix of derivatives) of the student loss w.r.t *w*, and $J_{\alpha}\left( i \right)$– the Jacobian of the teacher loss w.r.t *α* at input *i*, and *N* is the size of the minibatch.

## RP3Net implementation and training

RP3Net was implemented with PyTorch (Paszke *et al.* 2019). Foundation models were downloaded from HuggingFace (Wolf *et al.* 2020). Training loop was implemented with PyTorch Lightning (Falcon and The PyTorch Lightning team 2024). For models C and D, when the foundation model weights were fine-tuned during training, low-rank adaptation (LoRA) was used (Mangrulkar *et al.* 2022; Yu *et al.* 2023). Early stopping criterion was used, where training is terminated if AUROC for the validation dataset does not improve for 10 epochs. The exact revisions of software packages and foundation models, as well as training run configurations with hyperparameter values, are available in the RP3Net GitHub Repository.

## Experimental procedures for construct expression.

All sequences were codon optimised for *E. coli* using the proprietary GeneArt codon optimization algorithm provided by Thermo Fisher Scientific. The same codon usage was used for His-tags across constructs. The constructs were synthesized as synthetic genes and cloned into backbone vector pET24a. One construct failed during the cloning process. For small-scale soluble expression screening, the plasmid DNA was transformed into competent phage resistant *E. coli* BL21(DE3) cells (New England Biolabs #C2527H) in 96-well PCR plates. The transformation mix was used to directly inoculate 3mL LB media supplemented with 100ug/mL kanamycin in 24 deep-well plates and left shaking at 37°C overnight. Protein expression was auto-induced in rich ZYP-8012 media supplemented with 100ug/mL kanamycin in 24 deep-well plates, by inoculating 3mL with 50uL pre-culture and left shaking for 3 h at 37°C followed by 24 h at 18°C. After harvest (4000xg, 5min, 4°C), the pellets were lysed with 900uL lysis buffer (40mM HEPES, 300mM NaCl, 5mM imidazole, 10% glycerol, 1mM TCEP, 0.1% DDM, 0.2mg/mL lysozyme, DNAse & protease inhibitors) and freeze-thawed once. The lysate was cleared by centrifugation (4000xg, 30min, 4°C) before subjecting to a one-step Nickel affinity purification using an automated bead-based platform. The protein was captured on the magnetic beads for 30min at 4°C, followed by two wash steps to wash off unbound proteins (40mM HEPES, 300mM NaCl, 5mM imidazole, 10% glycerol, 1mM TCEP) and eluted in 100uL elution buffer (40mM HEPES, 300mM NaCl, 300mM imidazole, 10% glycerol, 1mM TCEP). 10uL of the elution was loaded onto NuPAGE Bis- Tris gels (Invitrogen) together with Novex Pre- stained protein marker (Invitrogen) and 5ug of an internal standard protein. The gels were stained in Der Blaue Jonas (GRP) and analysed using the densitometry software Image Lab (BioRad). The “Passed”, “Not Passed” and “Ambiguous” outcome annotations were provided manually by the lab scientist, based on the relative thickness and brightness of gel bands. Annotations from two separate biological replicates, along with the construct protein sequences and model scores, are shown in supplementary Table S2. The nucleotide sequences of the plasmid vectors in GenBank format are available on the FTP site (see Data Availability in the main text). Gels from both experiments are shown in Figure S3. The best outcome from the two experimental runs was used as the ground truth for the model evaluation.

# Supplementary figures


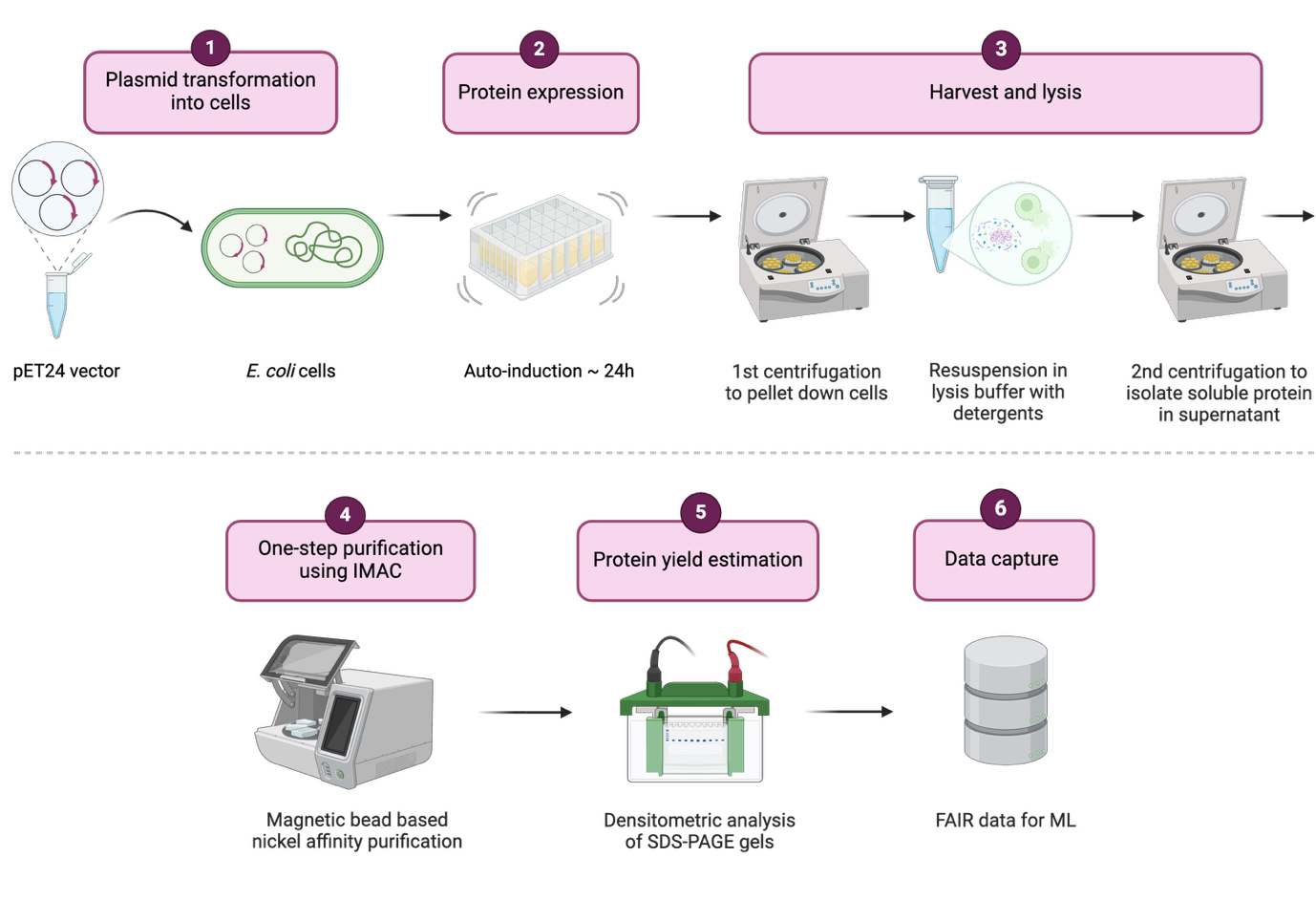


#### Figure S1

The experimental workflow for small-scale recombinant soluble protein production with one-step purification. After cloning, plasmids with the genetic material of the protein of interest are transformed into *E. coli* cells (step 1). The cells are grown for 24 hours (step 2). Harvesting involves two centrifugation steps: first to spin down cells, then, after lysis, to isolate the soluble protein in the supernatant (step 3). This is followed by IMAC purification (step 4), yield estimation via densitometric analysis of SDS-PAGE gels (step 5) and, finally, data capture for further analysis and machine learning (ML, step 6). Image generated with BioRender.com.

A


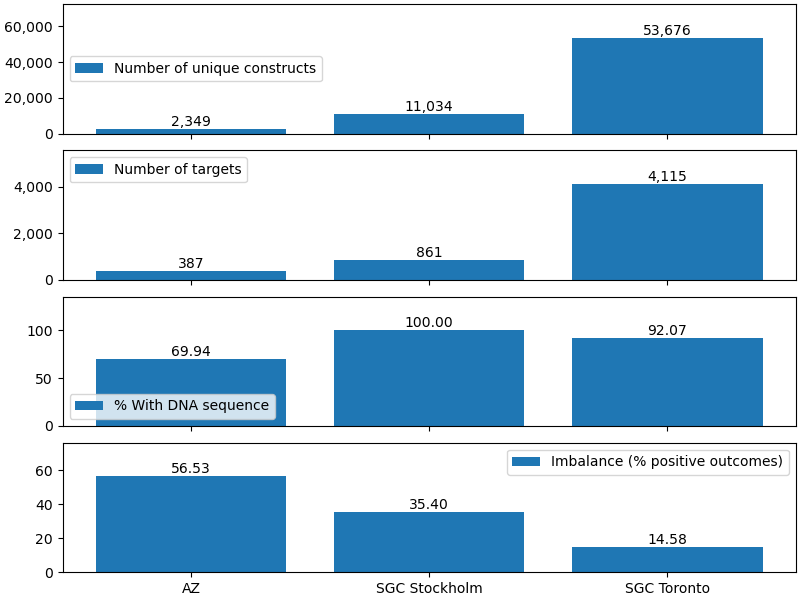


B


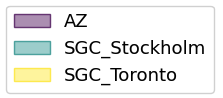

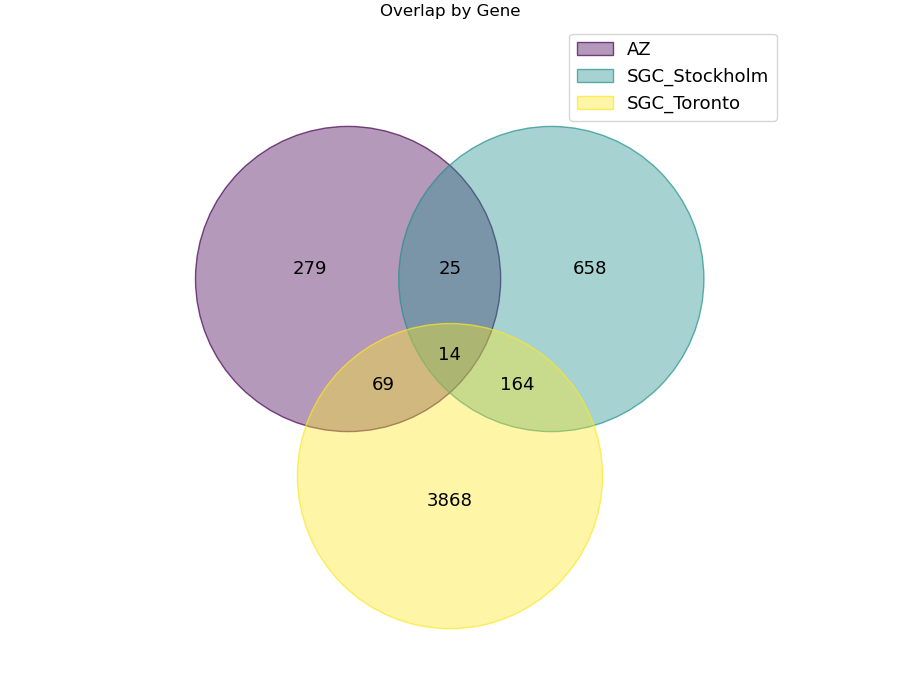


#### Figure S2.

The dataset overview. A: Numerical summary of the data by source, including number of unique constructs, number of targets, availability of DNA sequences and imbalance between positive and negative expression outcomes. B: Overlap between data sources, by target gene. Target genes were identified by searching the construct sequences against Uniref100 database (Suzek *et al.* 2015; Bateman *et al.* 2023) with DIAMOND v2.1.9 (Buchfink, Reuter and Drost 2021). The Uniref100 database was downloaded on the 18^th^ of July 2024, and the Uniparc sequences were removed from it.

#### Figure S3 – see separate Microsoft PowerPoint file.

SDS-PAGE analysis of the affinity-purified soluble fraction of the validation set in *E. coli* in biological replicates. Equal volume (10uL per lane) of the elution fraction from nickel affinity purification for the 97 proteins in the validation set was loaded onto a 4- 12% Bis- Tris SDS-PAGE gel in two biological replicates. Lane M: molecular weight marker; Lane S: internal protein standard (5ug). The gels were stained to visualise proteins. The black arrows indicate where the bands are expected based on the His-tagged protein’s respective theoretical molecular weight. Based on the relative intensity compared to the background, the outcome was annotated manually as either “Passed” (higher than background), “Ambiguous” (same as background) or “Not Passed” (lower than background). Degradation products in trimmed constructs (lanes 1-8) are indicated with dashed arrows. The full list of annotated experiment results, along with lane numbers, construct sequences and model scores is given in a separate ***Table S2,*** see separate files in Microsoft Excel and Apache Parquet formats.


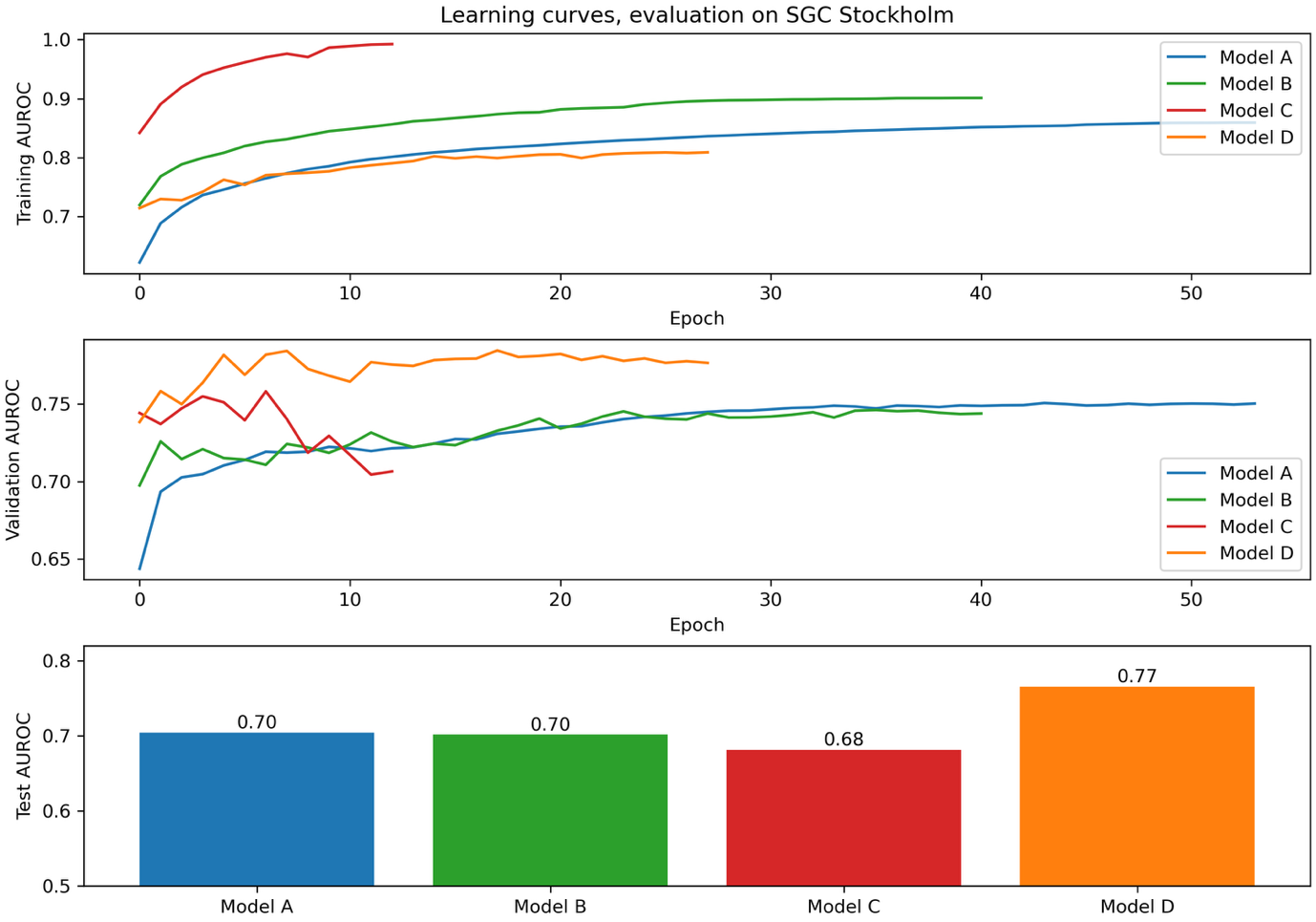


#### Figure S4.

Learning curves for Models A, B, C and D, evaluated on SGC Stockholm data. Model A is trained on SGC Stockholm data only. Models B, C and D are trained on the full data set. The model weights that achieve the highest validation AUROC were used for evaluation on the test dataset.

# Supplementary tables

#### Table S1.

Foundation models

| Model | Type | Architecture | Training Data Sources | Training Data Size | Number of Parameters | Comments |
| --- | --- | --- | --- | --- | --- | --- |
| ESM2 650M (Lin *et al.* 2023) | Protein | Transformer | UniRef50, UniRef90 | 65M | 650M |  |
| ESM2 3B (Lin *et al.* 2023) | Protein | Transformer | UniRef50, UniRef90 | 65M | 2.8B |  |
| ESM3 (Hayes *et al.* 2025) | Protein | Transformer | UniRef, MGnify, JGI IMG/M, OAS | 2.78B | 1.8B | Licensing restrictions apply |
| ESMC 600 (ESM Team 2024) | Protein | Transformer | UniRef, MGnify, JGI IMG/M | 2.3B | 575M | Licensing restrictions apply |
| ESMC 300 (ESM Team 2024) | Protein | Transformer | UniRef, MGnify, JGI IMG/M | 2.3B | 333M | Licensing restrictions apply |
| ProtT5 XL (Elnaggar *et al.* 2022) | Protein | Transformer | UniRef50 | 49M | 1.2B | Encoder only |
| ProtBert (Elnaggar et al. 2022) | Protein | Transformer | UniRef100 | 217M | 420M |  |
| ProteinBert(Brandes *et al.* 2022) | Protein | Convolutions + global attention | UniRef90 | 106M | 16M |  |
| HyenaDNA Medium(Nguyen *et al.* 2023) | DNA | Hyena | Human Genome hg38 | 3.2B bp | 24M | Maximum sequence length = 450K bases |
| HyenaDNA Large (Nguyen *et al.* 2023) | DNA | Hyena | Human Genome hg38 | 3.2B bp | 46M | Maximum sequence length = 1M bases |
| DNABert (Zhou *et al.* 2023) | DNA | Transformer | Genomes from 136 species belonging to 6 classes | 32.49B bp | 117M |  |
| CaLM (Outeiral and Deane 2024) | Codon | Transformer | European Nucleotide Archive, coding sequences | 8.7M | 85M |  |

# References

Bateman A, Martin MJ, Orchard S *et al.* UniProt: the Universal Protein Knowledgebase in 2023. *Nucleic Acids Res* 2023;**51**:D523–31.

Brandes N, Ofer D, Peleg Y *et al.* ProteinBERT: a universal deep-learning model of protein sequence and function. *Bioinformatics* 2022;**38**:2102–10.

Buchfink B, Reuter K, Drost HG. Sensitive protein alignments at tree-of-life scale using DIAMOND. *Nat Methods* 2021;**18**:366–8.

Elnaggar A, Heinzinger M, Dallago C *et al.* ProtTrans: Toward Understanding the Language of Life Through Self-Supervised Learning. *IEEE Trans Pattern Anal Mach Intell* 2022;**44**:7112–27.

ESM Team. *ESM Cambrian: Revealing the Mysteries of Proteins with Unsupervised Learning.* https://www.evolutionaryscale.ai/blog/esm-cambrian (January 16, 2025, date last accessed)

Falcon W, The PyTorch Lightning team. PyTorch Lightning. 2024.

Friedman JH. Greedy function approximation: A gradient boosting machine. *The Annals of Statistics* 2001;**29**, DOI: 10.1214/aos/1013203451.

Hayes T, Rao R, Akin H *et al.* Simulating 500 million years of evolution with a language model. *Science (1979)* 2025;**387**:850–8.

Jumper J, Evans R, Pritzel A *et al.* Highly accurate protein structure prediction with AlphaFold. *Nature* 2021;**596**:583–9.

Lin Z, Akin H, Rao R *et al.* Evolutionary-scale prediction of atomic-level protein structure with a language model. *Science (1979)* 2023;**379**:1123–30.

Linding R, Jensen LJ, Diella F *et al.* Protein Disorder Prediction. *Structure* 2003;**11**:1453–9.

Mangrulkar S, Gugger S, Debut L *et al.* PEFT: State-of-the-art Parameter-Efficient Fine-Tuning methods. 2022.

Mirdita M, von den Driesch L, Galiez C *et al.* Uniclust databases of clustered and deeply annotated protein sequences and alignments. *Nucleic Acids Res* 2016;**45**:D170–6.

Nguyen E, Poli M, Faizi M *et al.* HyenaDNA: Long-Range Genomic Sequence Modeling at Single Nucleotide Resolution. *ArXiv* 2023.

Outeiral C, Deane CM. Codon language embeddings provide strong signals for use in protein engineering. *Nat Mach Intell* 2024;**6**:170–9.

Paszke A, Gross S, Massa F *et al.* PyTorch: An Imperative Style, High-Performance Deep Learning Library. *Advances in Neural Information Processing Systems 32*. Curran Associates, Inc., 2019.

Sankar K, Trainor K, Blazer LL *et al.* A Descriptor Set for Quantitative Structure‐property Relationship Prediction in Biologics. *Mol Inform* 2022;**41**:2100240.

Steinegger M, Söding J. MMseqs2 enables sensitive protein sequence searching for the analysis of massive data sets. *Nat Biotechnol* 2017;**35**:1026–8.

Suzek BE, Wang Y, Huang H *et al.* UniRef clusters: a comprehensive and scalable alternative for improving sequence similarity searches. *Bioinformatics* 2015;**31**:926.

Taraday MK, Baskin C. Enhanced Meta Label Correction for Coping with Label Corruption. *ArXiv* 2023.

Wang S, Li W, Liu S *et al.* RaptorX-Property: a web server for protein structure property prediction. *Nucleic Acids Res* 2016;**44**:W430–5.

Wolf T, Debut L, Sanh V *et al.* Transformers: State-of-the-Art Natural Language Processing. *EMNLP 2020 - Conference on Empirical Methods in Natural Language Processing, Proceedings of Systems Demonstrations*. Association for Computational Linguistics (ACL), 2020, 38–45.

Yu Y, Yang C-HH, Kolehmainen J *et al.* Low-Rank Adaptation of Large Language Model Rescoring for Parameter-Efficient Speech Recognition. *2023 IEEE Automatic Speech Recognition and Understanding Workshop (ASRU)*. IEEE, 2023, 1–8.

Zheng G, Awadallah AH, Dumais S. Meta Label Correction for Noisy Label Learning. *Proceedings of the AAAI Conference on Artificial Intelligence (AAAI)*. 2021.

Zhou Z, Ji Y, Li W *et al.* DNABERT-2: Efficient Foundation Model and Benchmark For Multi-Species Genome. *ArXiv* 2023.

Zhu Y-H, Hu J, Ge F *et al.* Accurate multistage prediction of protein crystallization propensity using deep-cascade forest with sequence-based features. *Brief Bioinform* 2021;**22**, DOI: 10.1093/bib/bbaa076.
