# Supplementary material for: RP3Net: a deep learning model for predicting recombinant protein production in Escherichia coli
Source: Bioinformatics. 2026 Jan 11;42(1):btag003. doi: 10.1093/bioinformatics/btag003 (PMC12857573; doi:10.1093/bioinformatics/btag003)
Supplement: btag003_Supplementary_Data [file btag003_supplementary_data.zip › supplements/rp3net_fig_s3_experimental_validation_gels.pptx]

## Slide 1
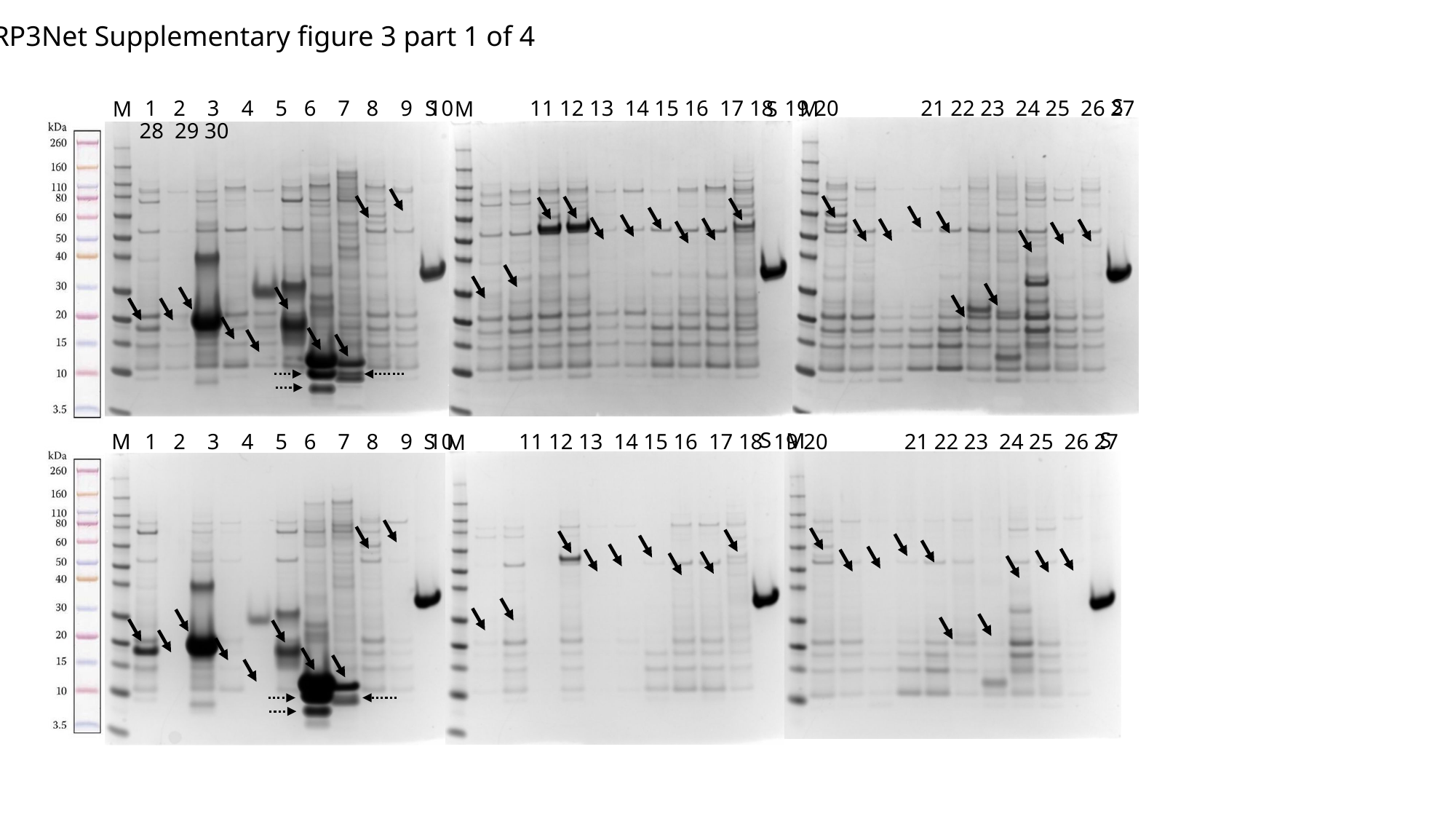

RP3Net Supplementary figure 3 part 1 of 4
S
S
 1 2 3 4 5 6 7 8 9 10 11 12 13 14 15 16 17 18 19 20 21 22 23 24 25 26 27 28 29 30
S
M
M
M
S
S
M
 1 2 3 4 5 6 7 8 9 10 11 12 13 14 15 16 17 18 19 20 21 22 23 24 25 26 27 28 29 30
M
S
M

## Slide 2
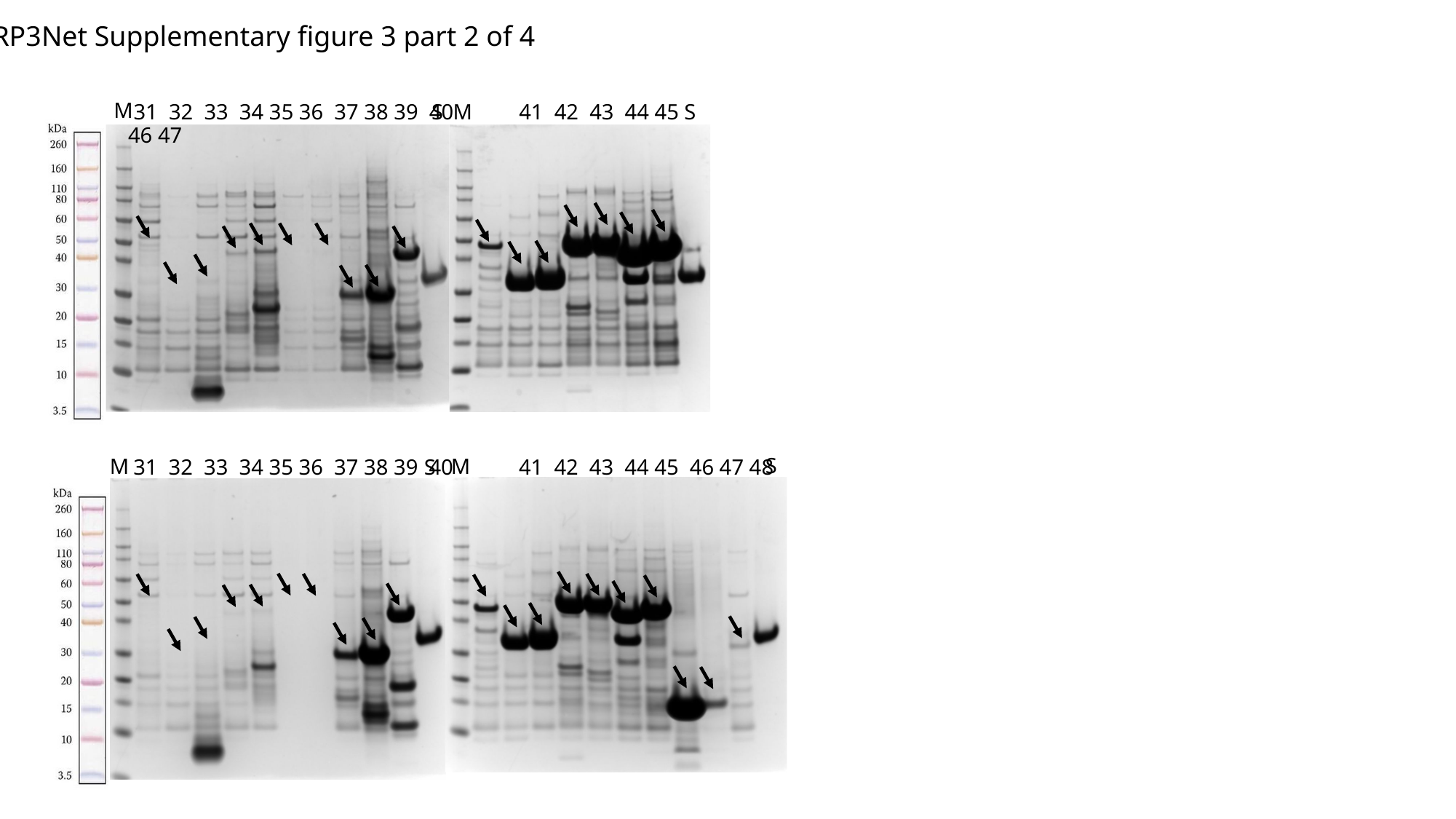

RP3Net Supplementary figure 3 part 2 of 4
M
S
 31 32 33 34 35 36 37 38 39 40 41 42 43 44 45 46 47
M
S
S
M
M
 31 32 33 34 35 36 37 38 39 40 41 42 43 44 45 46 47 48 49 50
S

## Slide 3
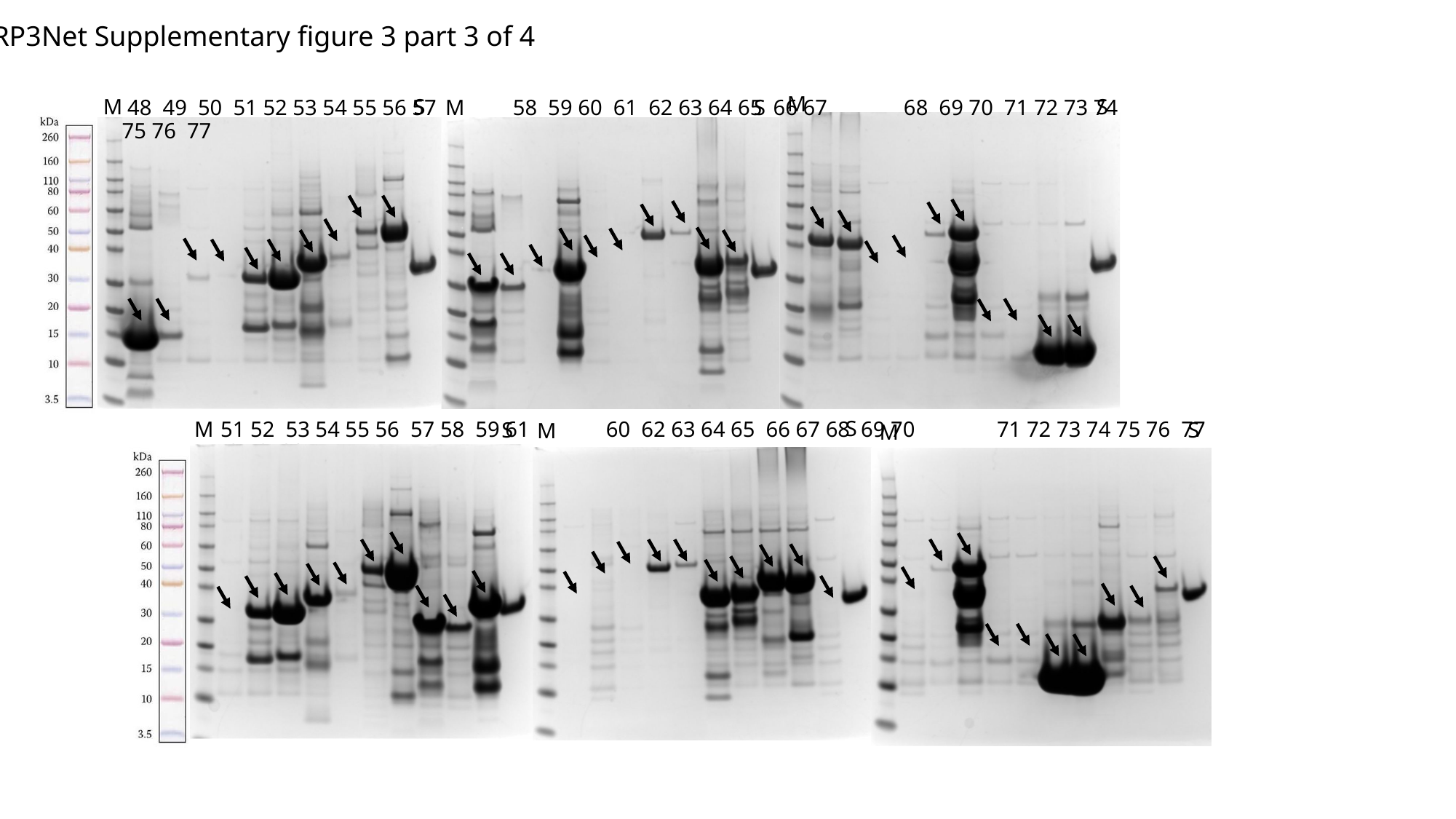

RP3Net Supplementary figure 3 part 3 of 4
M
M
S
S
S
 48 49 50 51 52 53 54 55 56 57 58 59 60 61 62 63 64 65 66 67 68 69 70 71 72 73 74 75 76 77
M
S
 51 52 53 54 55 56 57 58 59 61 60 62 63 64 65 66 67 68 69 70 71 72 73 74 75 76 77 78 79 80
M
S
S
M
M

## Slide 4
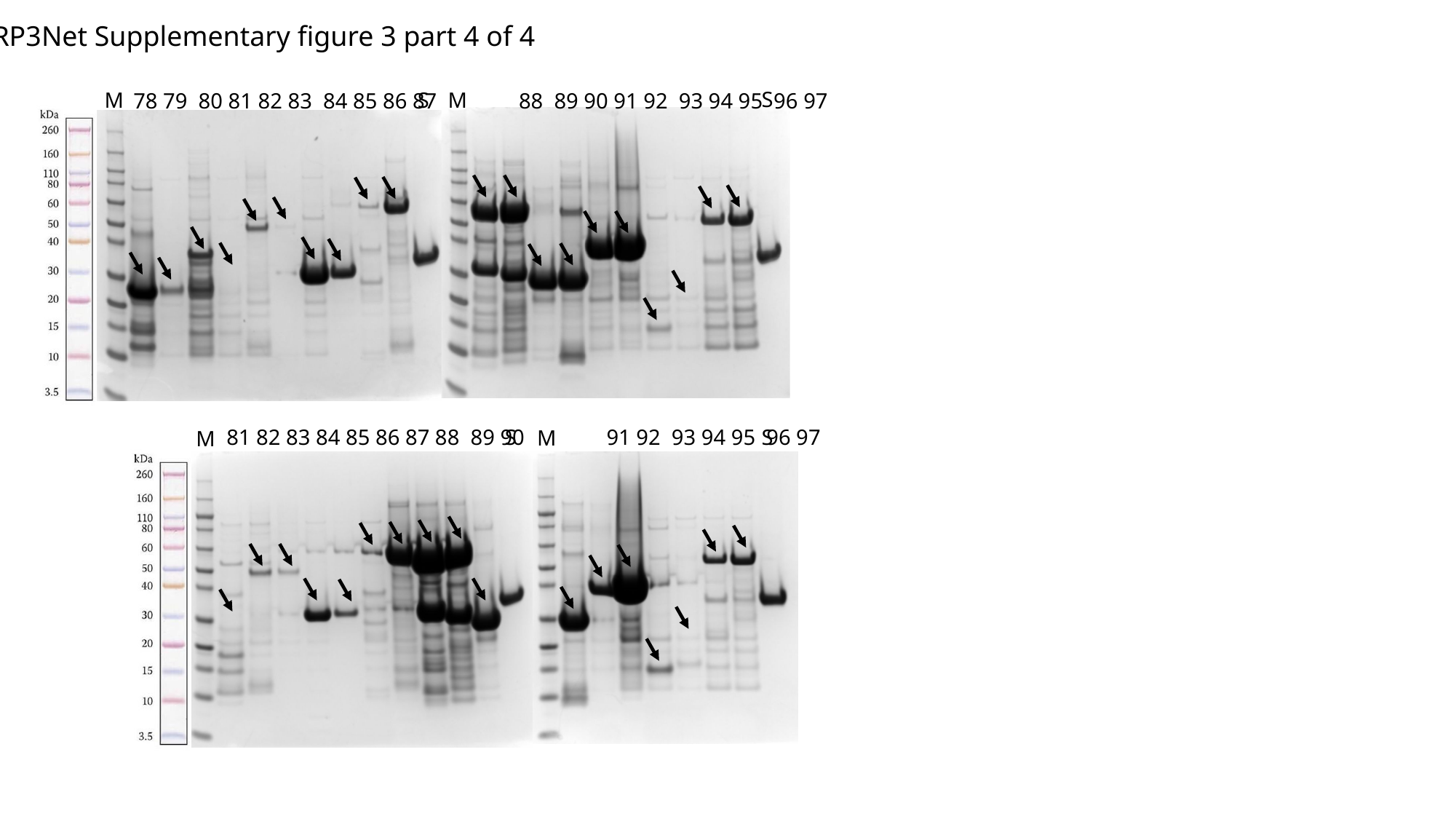

RP3Net Supplementary figure 3 part 4 of 4
S
M
S
M
 78 79 80 81 82 83 84 85 86 87 88 89 90 91 92 93 94 95 96 97
 81 82 83 84 85 86 87 88 89 90 91 92 93 94 95 96 97
S
S
M
M
